# Live brain biopsy during CSF shunt surgery for precision diagnostics and multi-omic research

**DOI:** 10.1186/s13195-026-02150-0

**Published:** 2026-08-12

**Authors:** Heikki E. M. Lukkarinen, Antti J. Luikku, Antonios G. Dougalis, Anssi Pelkonen, Mireia Gomez-Budia, Polina Abushik, Nataliia Novosolova, Henna Jäntti, Antti-Pekka Elomaa, Aku Kaipainen, Nikolai Klimko, Paula Walle, Henna-Kaisa M. Jyrkkänen, Nils Danner, Katri Piilonen, Nora E. Firtser, Arto Immonen, Juha E. Jääskeläinen, Ross W. Paterson, Kristopher T. Kahle, Per Kristian Eide, Evan Macosko, Beth Stevens, Valtteri Julkunen, Eino Solje, Mari Takalo, Sini-Pauliina Juopperi, Susanna Kemppainen, Henna Martiskainen, Anssi Lipponen, Mikko Hiltunen, Tuomas Rauramaa, Tarja Malm, Ville Leinonen

**Affiliations:** 1 NeuroCenter, Neurosurgery, KUH Kuopio University Hospital, P. O. Box 100, Kuopio, FI-70029 KYS Finland; 2 Neurosurgery, Institute of Clinical Medicine, University of Eastern Finland, Kuopio, 70211 Finland; 3 Stanley Center for Psychiatric Research, Broad Institute of MIT and Harvard, Cambridge, MA 02142 USA; 4 A. I. Virtanen Institute for Molecular Sciences, University of Eastern Finland, Kuopio, 70211 Finland; 5 Department of Neurosurgery, University of Helsinki and Helsinki University Hospital, Helsinki, 00290 Finland; 6 Department of Neurodegenerative Diseases, UCL Queen Square Institute of Neurology, London, WC1N 3BG UK; 7 Department of Neurosurgery, Harvard Medical School and Massachusetts General Hospital, Boston, MA 02114 USA; 8 Broad Institute of MIT and Harvard, Cambridge, MA 02142 USA; 9 Department of Neurosurgery, Oslo University Hospital-Rikshospitalet, Oslo, 0372 Norway; 10 Institute of Clinical Medicine, Faculty of Medicine, University of Oslo, Oslo, 0372 Norway; 11 K.G. Jebsen Centre for Brain Fluid Research, University of Oslo, Oslo, 0372 Norway; 12 Department of Psychiatry, Massachusetts General Hospital, Boston, MA 02114 USA; 13 Department of Neurobiology, Harvard Medical School, Boston, MA 02115 USA; 14 F.M. Kirby Neurobiology Center, Boston Children’s Hospital, Boston, MA 02115 USA; 15 Harvard Medical School, Boston, MA 02115 USA; 16 Howard Hughes Medical Institute, Boston Children’s Hospital, Boston, MA 02115 USA; 17 Neurology, Institute of Clinical Medicine, University of Eastern Finland, Kuopio, 70211 Finland; 18 NeuroCenter, Neurology, Kuopio University Hospital, Kuopio, 70029 Finland; 19 Institute of Biomedicine, University of Eastern Finland, Kuopio, 70210 Finland; 20 Unit of Pathology, Institute of Clinical Medicine, University of Eastern Finland, Kuopio, 70211 Finland; 21 Department of Pathology, Kuopio University Hospital, Kuopio, 70029 Finland

**Keywords:** Cortical brain biopsy, Alzheimer’s disease, Idiopathic normal pressure hydrocephalus, Single-cell and spatial omics, Electrophysiology

## Abstract

Diagnostic testing and neurosurgical management involving CSF shunting in patients with idiopathic normal pressure hydrocephalus (iNPH) offers a unique, ethically sustainable opportunity to study the aging human brain. The common co-occurrence of neurodegenerative pathologies such as amyloid-β plaques and neurofibrillary tau tangles (NFT) provides a valuable window for research into the Alzheimer’s disease-related mechanisms. Interindividual variability among iNPH patients enables investigation and validation of biomarkers and molecular mechanisms, opening possibilities for new treatments. In vitro techniques and culturing methods for living brain tissue expand the utility of samples for electrophysiological studies, drug testing, and examining genetic risk variants. Careful sectioning and processing are essential for high-quality samples with intact cells for functional analysis. A rigorous pipeline is also needed for preserving samples for transcriptomic, proteomic, and structural analyses, with emphasis on RNA integrity and tissue architecture for single-cell sequencing and spatial analysis. Here, we review the use of small cortical brain biopsies in neurodegenerative diseases research and diagnostics. Furthermore, we share our team’s experience in acquiring and preserving living brain samples during iNPH CSF shunt surgeries, along with our protocols for resection, use, and preservation of biopsies for preclinical research. We highlight the potential of living human tissue for neuroscience, early neurodegenerative disease diagnosis, and targeted therapies.

## Introduction

Precise diagnostics for human protein-accumulating degenerative brain diseases are still delayed until the neuropathological assessment of affected brain tissue is possible. Despite advances in biological surrogate marker profiling in living humans, which clarify the diagnosis during life, research into disease mechanisms at the cellular and molecular levels remains mostly limited to postmortem brain tissue, which virtually reflects only the end-stage of the disease or to cell cultures and organoids [1–3]. Future research and care are projected to become more patient-centered and obtaining living human samples for research purposes is part of this initiative [4–6]. Emerging technologies and research methods in genomics, lipidomics, transcriptomics, and single-cell imaging, combined with spatial information and AI-driven data management and analysis protocols, will be essential to this effort. These approaches are expected to reveal previously unknown cell- and tissue-level disease processes in complex neurological and neurodegenerative diseases [2, 6]. A fresh brain tissue sample from a living human is likely the closest to replicate the in vivo mechanisms of the affected living brain. When properly preserved, it can be used for genomic analysis, immunohistochemistry (IHC), morphological analysis, and electrophysiological recordings [3, 7–12], enabling a multimodal research approach. Epilepsy and neuro-oncology procedures are the most well-known and reported opportunities for obtaining these samples [4, 5]. In neuro-oncology, biobanking resected brain tissue and other readily available tissue samples has become recommended practice [4]. However, brain surgery is rarely necessary for neurodegenerative brain disorders. The current consensus restricts diagnostic brain biopsies in common neurocognitive diseases and clinical neurological practice to situations where a specific diagnosis is essential and cannot be determined by other means, and the suspected condition is considered treatable [13, 14]. Overall, the literature on the feasibility, safety, and justification of brain biopsy in non-neoplastic neurological and neurodegenerative brain diseases is inconsistent and limited. However, idiopathic normal pressure hydrocephalus (iNPH) is an exception, as it is a neurological disorder in which symptoms can be alleviated with CSF shunt surgery [15]. This creates a feasible and ethically sustainable window to collect brain samples valuable for more precise diagnostics and neurodegenerative brain disorder research.

Our aim is to review the current literature on how cortical brain samples from patients with clinically diagnosed iNPH can offer a potential model for studying in vivo neurodegenerative disorders and disease-affected brain physiology. Additionally, maintaining the viability, cellular morphology, and cytological structure of brain tissue samples for analysis involves methodological challenges. Therefore, we describe our Kuopio University Hospital (KUH, Kuopio, Finland) Department of Neurosurgery research protocol for obtaining high-quality brain biopsies during CSF shunt surgery in iNPH patients and outline the ongoing collaborating research programs collecting ex vivo brain tissue in iNPH globally.

## Methods

### Literature review

This narrative literature review was conducted in accordance with Preferred Reporting Items for Systematic Reviews and Meta-Analyses (PRISMA) guidelines [16] where applicable. A manual search of original articles related to brain biopsy was performed in PubMed and Google Scholar using specific search strings. The search strings included “Brain biopsy” AND “dementia,” “normal pressure hydrocephalus,” “Alzheimer’s disease,” and “transcriptomics” (412 results). The search spanned from January 1960 to January 2025 and aimed to identify original publications on brain biopsy in non-neoplastic neurological disorders and their applications.

### Study population for safety reporting

Additional patient characteristics and cause-of-death records were collected for NPH patients treated with CSF shunt surgery at Kuopio University Hospital, Kuopio, Finland. All available cases between January 1991 and April 2025 were included. Baseline age, sex and 12-month postoperative follow-up cause-of-death records were obtained. Surgery-related causes of death were determined using ICD-10 codes from death certificates, complication-related CT images and clinical health records. Statistical analyses were conducted using R 4.4.2 [17]. Age comparison was made with independent samples t-test. Proportions were compared using a two-sided Fisher’s exact test, and 95% confidence intervals were estimated using the Clopper-Pearson method.

## Results

### Literature review

#### Idiopathic normal pressure hydrocephalus (iNPH)

Idiopathic normal pressure hydrocephalus (iNPH) is a degenerative brain disease primarily affecting individuals in their 70 s and 80 s [18, 19]. The etiology likely involves macro- and microscopic impairments in CSF dynamics within the brain that may lead to reduced periventricular metabolism [20–24]. Its symptoms are potentially alleviable with a neurosurgically inserted CSF shunt [15, 25]. Hallmark neuropathological changes of Alzheimer’s disease (AD), such as amyloid-β plaque (Aβ) pathology, are seen in 30–68% of iNPH patients’ cortical brain biopsies, which can be collected during the procedure. Both Aβ plaque and NFTs appear in 7–22% of these biopsies [7, 26–38]. Even higher prevalences of 63% and 49%, respectively, have been reported in an older cohort [39]. The prevalence and severity of AD and NFT pathology are similar to those seen in unselected autopsy cohorts in corresponding age-groups [40]. However, clinical AD diagnoses were 40% more frequent in shunted iNPH patients as compared to age-matched participants [29]. The frequent and varying burden of AD pathology in age-diverse iNPH cohorts demonstrates the natural progression of AD [7, 28, 31, 32, 34–36, 39, 41–43], making the biopsies obtained during shunt surgery an ideal material for studying early AD. Importantly, these biopsies cover the entire pathological spectrum, from sparse, fleecy Aβ plaques to widespread, dense plaques, along with varying amounts of NFTs and controls with no pathology [7, 26, 33, 34, 39]. Furthermore, coexisting AD in iNPH patients is considered a true comorbid condition with the same known risk factors and identical neuropathological changes; it is not a distinct subgroup of either AD or iNPH [24, 28, 33, 39, 43–45]. INPH also features a partially AD-like CSF biomarker profile, including low levels of Aβ42 [24] and has AD-related genetic variants, both predisposing and protective ones, occurring at approximately the expected frequency [23, 45]. The AD pathology-related findings in iNPH patients may therefore be applicable to non-iNPH AD populations [39], although further validation in independent cohorts is needed.

#### Brain biopsy in diagnosis and prognostication

In neuro-oncology, tumor biopsies play a routine role in diagnostics, therapeutics and prognostication [46, 47]. Furthermore, in carefully assessed patients with non-neoplastic diseases, a timely brain biopsy with rigorous neuropathological analysis can provide a definitive diagnosis in up to 50–80% of cases [14, 48–52]. In a retrospective single-center study*,* 178 patients with non-neoplastic neurological disorders underwent brain biopsies either from a specific lesion (*n* = 127) or non-lesioned parenchyma (*n* = 51). A diagnosis was achieved in 83% of cases, most commonly inflammatory diseases (22%), infections (20%), and hematological malignancies (20%) [52]. However, diagnostic yield was lower (46%) in patients without visible lesions [52]. Notably, arguments for limited diagnostic value in neurocognitive diseases or those with non-neoplastic, unknown etiology neurological diseases have also been presented [13, 14]. In simulated brain biopsies of autopsy patients, cortical samples (1 cm^3^) enabled accurate diagnosis of the most common neurodegenerative brain disorders (Table 1), consistent with full postmortem neuropathological assessments of the brain [53, 54]. The two studies found that frontal lobe biopsies were relatively accurate, with sensitivities of 64% and 81% and specificities of 43% and 79% for detecting AD, corticobasal degeneration, multisystem atrophy, Lewy body disease, frontotemporal lobar degeneration with TDP-43, Huntington’s disease, cerebrovascular disease, and controls. However, they poorly detected progressive supranuclear palsy and amyotrophic lateral sclerosis (Table 1) [53, 54]. These studies also reported high inter-rater agreement between neuropathologists, corroborating the usability of smaller brain biopsies in neuropathological examination. In suspected cases of Creutzfeldt-Jakob’s disease, cortical brain biopsies confirmed diagnosis in 42% of patients [50], suggesting that biopsies can potentially be useful after non-invasive diagnostics (MRI, CSF, EEG) have been conducted. In a cohort of patients with unknown dementia, Creutzfeldt-Jakob’s disease was the second most diagnosed condition (12%) after AD, when brain biopsies were performed [13].

**Table 1:** Anticipated sensitivity of frontal cortex brain biopsy in neurocognitive diseases

**Diagnosis**	**Number**	**Sensitivity**	Study*
Control	16	Na	Venneti et al
AD	25	80–100%	Venneti et al./King et al
PSP	12	0%	Venneti et al./King et al
CBD/MAPT	13	66–100%	Venneti et al./King et al
LBD	24	60–79%	Venneti et al./King et al
MSA	10	71–100%	Venneti et al./King et al
HD	6	83%	King et al
FTLD-TDP/MND	15	67–88%	Venneti et al./King et al
ALS	2	0%	King et al
PiD	6	66%	Venneti et al
CVD	1	100%	King et al

legend: Comparison of simulated biopsy and neuropathological examination in autopsy cohorts presented in *King et al*.* [53], Venneti et al. [54]

*AD* Alzheimer’s disease, *ALS* amyotrophic lateral sclerosis, *CBD/MAPT* corticobasal degeneration/microtubule-associated protein tau gene, *CVD* cerebrovascular disease, *FTLD-TDP/MND* frontotemporal lobar degeneration with TDP-43 pathology/with motor neuron disease, *HD* Huntington’s disease, *LBD* Lewy body disease, *MSA* multiple system atrophy, *PiD* Pick’s disease/behavioral variant of Frontotemporal dementia, *PSP* progressive supranuclear palsy

**Fig. 1:**
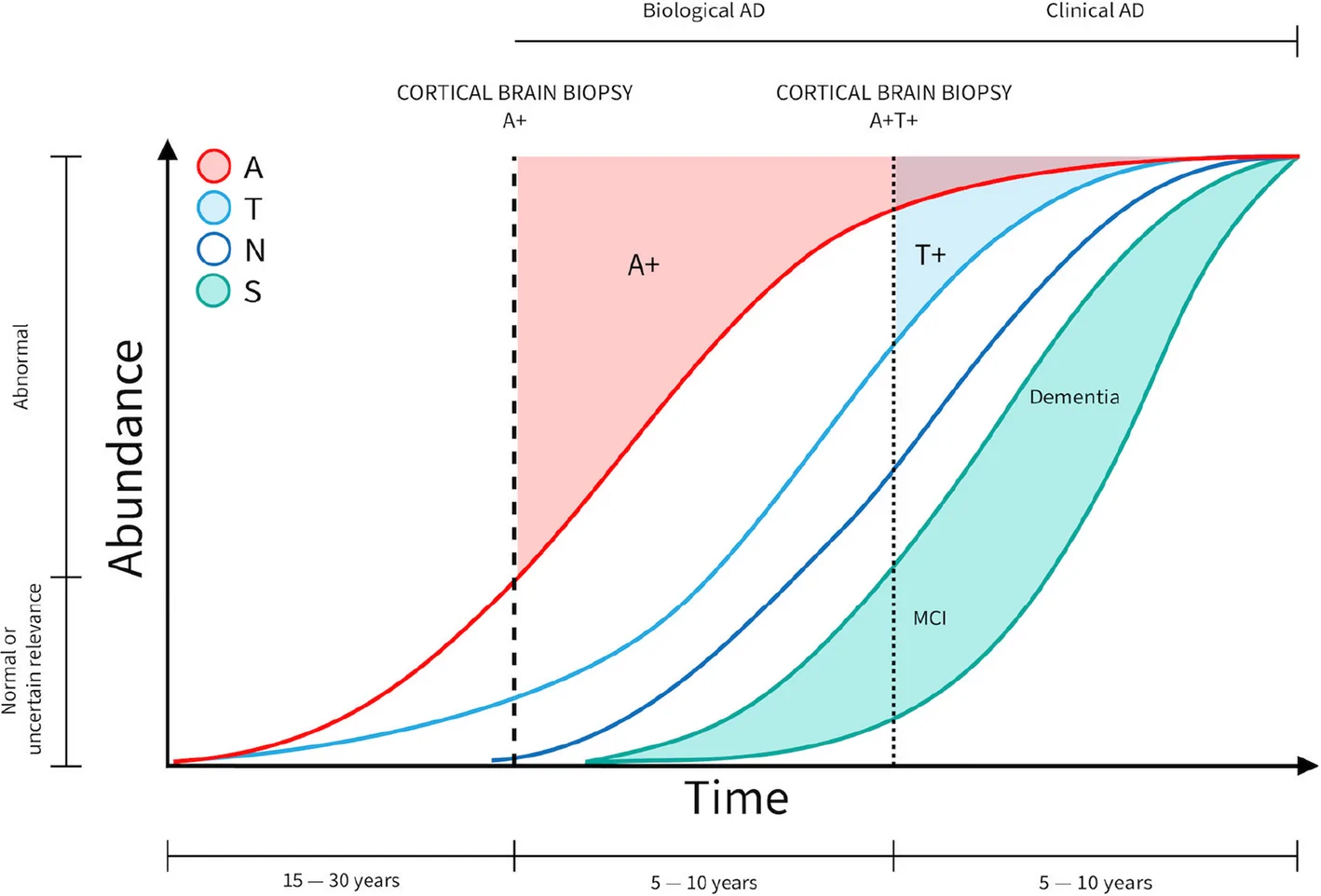
The schematic figure of cortical brain biopsy in iNPH patients in the context of the Alzheimer’s Disease continuum. Here the classical hypothesis of Alzheimer’s disease pathogenesis [59] involving amyloid-β (A) and tau (T) proteins is illustrated. The red (A), light blue (T) and dark blue (N) lines represent the abundance of ATN-classified pathologies over time. The turquoise area indicates the clinical manifestation of AD with its upper and lower boundaries reflecting individual differences in resilience. The vertical cut points mark the earliest time at which a brain biopsy would test positive for amyloid alone or for both amyloid and tau pathologies. The light red and light blue shaded areas adjacent to these cut points represent the time spans during which A and T positivity can be detected based on neuropathological findings from brain biopsies. A, amyloid- β plaque pathology; AD, Alzheimer’s disease; MCI, mild cognitive impairment; N, neurodegeneration; S, symptoms; T, neurofibrillary tau tangle pathology

Under National Institute on Aging and Alzheimer’s association (NIA-AA) neuropathological guidelines for Alzheimer’s disease, the use of surgical specimens e.g., brain biopsies obtained during operations is supported [55]. The guideline, however, recognize the limits of a small tissue sample in the regional evaluation of pathology. In frontal cortex brain biopsies, a significant presence of Aβ plaques indicates at least Thal phases 1 or 2, and NFT pathology corresponds to Braak stage V or VI [55–57]. According to the biological definition and the Aβ deposition (A), pathologic tau (T), and neurodegeneration (N) (ATN-) classification-based AD continuum hypothesis, A + individuals reflect early-stage AD, while A + T + individuals represent intermediate to advanced stages of AD (Fig. 1) [38, 39, 58, 59]. Due to the surgical management in iNPH, several studies have assessed brain biopsies in these patients for comorbid AD. Overall, an A + or A + T + cortical brain biopsy strongly predicts the development of clinical AD within a 2–5 year follow-up in elderly individuals [7, 45, 58, 60] and in diagnosed iNPH patients [29, 32, 36, 41]. However, in shunt-responsive iNPH patients, the effect is smaller [61]. During a 4-year follow-up, 33% of patients with an initially A + brain biopsy developed clinical AD, whereas only 5% of those with pathology-free brain biopsies developed clinical AD [60]. In diagnosed iNPH patients, 8–47% develop clinical AD during a 2–5-year follow-up. However, about 30–70% of these patients initially had brain biopsies free of AD neuropathological changes [7, 29, 32, 36, 60, 61]. Furthermore, AD neuropathological changes do not predict other neurocognitive diseases or MCI [60], and their absence correlates with normal Clinical Dementia Rating scores [58]. Similarly, the A + or A + T + brain biopsy correlates with lower Mini-Mental State Examination scores and reduced cognition in most studies [7, 26, 31, 33]. Additionally, in studies with iNPH patients, Aβ pathology in the frontal cortical biopsies correlate well (*r* = 0.82) with postmortem evaluations [28] and is equally or more prevalent at autopsy [62]. Regarding shunt surgery prognosis, extensive AD-related pathology is a risk factor for symptom irreversibility [34, 36, 37], although improvement can still occur in patients with comorbid AD [26, 33, 41, 60, 63]. It remains unclear whether a specific amount of Aβ and NFT pathologies is enough to exclude patients from shunt surgery. The overall average survival of presumed NPH patients is 6.3 years and is not influenced by biopsy A or AT positivity, but dementia-related causes of death are more common in A + T + individuals [64]. In general, common AD risk factors such as age and female gender similarly increase the likelihood of A + cortical brain biopsy [58, 60].

However, determining the cause of neurocognitive decline in shunted iNPH patients is a diagnostic challenge. It is important to recognize that other than AD-related or multiple brain pathologies may also contribute to cognitive decline. Some A-T- individuals who develop dementia are most likely affected by either the limited size of the brain biopsy and its inability to detect patchy pathological changes, or by other neurodegenerative disorders such as Lewy body disease or limbic-predominant age-related TDP-43 encephalopathy (LATE) [65]. Furthermore, based on autopsy studies, up to 10% of iNPH patients may eventually have progressive supranuclear palsy or corticobasal degeneration [27, 28, 62], potentially representing the A-T + group in biopsy studies. Another common finding in iNPH is vascular changes that may be seen in 25% of brain biopsies [63] and up to 70% in autopsied brains [28]. Therefore, part of the secondary dementia after shunt surgery is likely explained by vascular etiology.

#### Safety profile of brain biopsy in non-neoplastic brain diseases

We identified 18 research articles covering 1,182 cases related to brain biopsy in non-neoplastic brain diseases, including both diagnostic biopsy procedures [13, 14, 48–50, 52] and additional brain biopsies performed during NPH shunt surgery or diagnostic ICP monitoring for NPH [20, 34, 42, 64, 66, 67]. The combined frequency of biopsy-related adverse events resulted in a complication rate of 3.2% (38 out of 1182) and a mortality rate of 0.7% (8 out of 1182). Most authors reported complications to be clinically significant but rarely resulting any long-lasting neurological sequalae [13, 49, 52, 60, 64]. Notably, combined rates for diagnostic brain biopsies alone, were 5.5% (*n* = 32) for complications and 0.9% (*n* = 5) for mortality, with substantial variation in sample size (up to 1 cm^3^), location (cortical, subcortical, deep brain, lesion-based) and surgical techniques (open or stereotactic) [13, 14, 48–50, 52]. The largest diagnostic biopsy study by Mathon et al*.* (*n* = 178) reported that 12.3% of patients to developed complications during the first postoperative month, most of which were asymptomatic changes seen in postoperative brain imaging [52]. The mortality rate with two deaths was 1.1%, and one patient (0.6%) developed permanent neurological morbidity. Most biopsies were deep brain (36%) or subcortical (41%) and over 1 cm^3^ in size (71%) [52]. Moreover, small cortical brain biopsies obtained during the invasive management of NPH had markedly lower combined procedure-related complication rate of 1.0% (6 out of 600) and mortality rate of 0.5% (3 out of 600) [20, 34, 42, 60, 67]. These biopsies included samples from the frontal, parietal and temporal cortex, and were all smaller in size (≤ 0.5 cm^3^). Of the three reported cases leading to mortality, only one occurred acutely while the other two were observed during 12-month follow-up. Compared to a large register study in Sweden (*n* = 979) reporting 0.5% 30-day mortality rate for iNPH shunt surgery [68], the additional risk from biopsy appears minimal. However, taking a brain biopsy without simultaneous treatment, such as shunting, is considered to have an increased risk–benefit ratio and is therefore limited to rare occasions following careful evaluation [13, 14].

#### Brain biopsy pathology associates with imaging- and fluid-based biomarkers

Several emerging imaging modalities have been developed to detect Aβ and NFT pathologies. However, studies comparing these modalities with neuropathology from living human brain biopsies remain limited. PET tracers for detecting fibrillary Aβ plaques in brain parenchyma typically include (11C)PIB [69], (18F)florbetaben, (18F)florbetapir [70] and (18F)flutemetamol [71]. Both (11C)PIB and (18F)flutemetamol PET uptake in the frontal cortex as well as neocortical uptake scores correlate well with histochemistry (Spearman’s ρ 0.6–0.7) and IHC Aβ load in cortical biopsies (ρ 0.4–0.8) [42, 72–74]. This supports the use of A + frontal cortex biopsies as surrogates for broader neocortical Aβ pathology, confirmed by the high correlation between biopsy site and the neocortex Aβ SUVR in (18F)flutemetamol PET studies [42, 74]. Novel tau-PET tracers, such as (18F)flortaucipir, have not yet been compared with biopsy neuropathology but demonstrate high sensitivity and specificity in distinguishing late-stage NFT pathology confirmed in autopsy (Braak V-VI) [75]. Tau-PET imaging is likely to align with biopsy findings as it closely resembles the hierarchical pattern of tau accumulation [76, 77]. However, alternative tau spreading patterns [56] beyond Braak staging may affect the sensitivity of cortical biopsies [78]. Similarly to biopsy-confirmed Aβ or Aβ/tau positivity, individuals with A + T + PET profiles are at high risk for AD related dementia, while A + individuals are at risk for mild cognitive impairment [79].

CSF- and blood-based biomarkers have made significant advances in recent decades. Particularly CSF markers are considered well-established for the diagnosis of AD pathology, along with novel markers for both diagnosis and prognosis in AD and other neurodegenerative brain diseases. The association between AD-related brain pathology and biomarker levels in blood or CSF is well studied in AD [80, 81], but less so in comparison to brain biopsy. Of the conventional AD CSF biomarkers, Aβ42 or the Aβ42/40 ratio reflects amyloid-PETs and illustrates the Aβ plaque pathology [81, 82]. CSF total tau (T-tau) and phosphorylated tau (P-tau181) have been considered markers of tau pathology [82]. However, T-tau also rises sharply in acute conditions affecting neuronal axons, such as stroke, head trauma, surgical procedures and Creutzfeldt Jakob’s disease and is therefore viewed more as a marker of neuroaxonal injury in recent research guidelines [38]. P-tau181 and other isoforms of 231 and 217 are the standard markers of tau related NFT pathology [80–82]. Still, the A and T markers are not exclusive to their respective pathologies, as both correlate with Aβ and tau pathologies in autopsy-based studies of AD and other neurocognitive diseases [81–84]. In plasma, Aβ42/40, P-tau217 and P-tau181 show the strongest correlation with Aβ plaques, while P-tau181 and especially P-tau217 also correlate with NFTs [81]. In Parkinson’s disease, α-synuclein assays in plasma or CSF are under investigations. However, it has not been studied via brain biopsy and is rarely compared to autopsy-confirmed pathology [85].

Patients with both iNPH and AD show a similar relationship between CSF biomarkers and brain pathology as observed in AD alone. Multiple studies report that A + and A + T + brain biopsies are linked to decreased Aβ42 and increased T-tau and P-tau181 levels in CSF [7, 26, 31, 32, 58, 86, 87]. However, a few smaller studies did not replicate these associations [30, 73]. Additionally, the amount of Aβ pathology in brain biopsies correlates with CSF Aβ42 levels [32, 58]. When predicting a brain biopsy AD pathology in iNPH patients, CSF levels of P-tau181, Aβ42 and their ratio in lumbar and ventricular samples are useful indicators. The Aβ42/P-tau181 ratio yields a receiver-operating characteristic area under curve (ROCAUC) of 0.7–0.8 [26, 31, 35, 58, 87]. For autopsy-confirmed pathology, the AUC improves to 0.9 [82]. An ongoing study at UCL is investigating the sensitivity and specificity of plasma biomarkers (p-tau217) for detecting Alzheimer pathology captured from a parietal biopsy.

#### Brain biopsy in genetics and transcriptomics

The field of neuro-oncology has paved the way for research on disease specific genomic changes. In particular, understanding somatic mutations and epigenomic profiles in brain tumors has significantly advanced neuropathological diagnostics and treatment strategies [2]. In non-neoplastic brain diseases, the genetic studies on living human brain tissue samples are relatively sparse but could be highly beneficial [2]. Somatic mutations or epigenetic alterations may play key roles in several neurosurgical conditions [2], as demonstrated in epilepsy patients with focal cortical dysplasia [5]. Moreover, transcriptomic research in AD has revealed multiple disease-related mechanisms. Bulk RNA sequencing, which captures the entire transcriptome of a specific tissue, has paved the way for both early and recent discoveries [1]. However, in complex neurological diseases, bulk sequencing lacks the resolution to identify more minor cell subpopulations or opposing gene expression patterns that may be critical to disease etiology and pathogenesis [10, 88]. For example, high-quality bulk RNA sequencing from AD samples failed to detect microglial changes that were revealed by single-cell RNA sequencing (scRNA-seq) [10].

A brain biopsy provides a window into exploring the implications of genes and their alleles in various brain disorders. This is illustrated by a study examining the overlap between type two diabetes (T2D) and AD in AT transgenic mice and iNPH patients [89]. The authors found abnormal microglia function related to weight and T2D in both models. RNA sequencing showed expression changes in genes like *PIK3R1* and *TREM2*, and they also identified altered downstream signaling associated with these genes [89]. Another example in comorbid iNPH and AD shows that carrying the *APOE* ε4 allele influences AT biomarkers, as ε4 allele increases both Aβ load and plaque burden, lowers CSF Aβ42 levels and increases P-tau181 levels [45, 58, 86, 90]. In contrast, the *APOE* ε2 allele is associated with higher CSF Aβ42 and lower P-tau181 [90]. Additionally, four other loci have been linked to AD-core markers [90], though further validation in brain tissue-based studies is needed to confirm and expand on these findings.

ScRNA and single-nucleus RNA (snRNA) sequencing are methods capable of exploring the cellular transcriptome of preserved tissue samples, including brain biopsies [7–11]. ScRNA sequencing depends on successfully releasing viable and intact cells from tissue, while snRNA sequencing is more versatile, allowing recovery of nuclei from both fresh frozen and formalin-fixed paraffin-embedded (FFPE) tissue samples [11, 91]. This flexibility enables more comprehensive cell-type analysis, including those susceptible to manipulation. In autopsied brain tissue from AD patients, scRNA sequencing has uncovered disease-related cellular disruptions in myelination, neuroinflammation, synaptic function, and neuron survival [8–10]. More recently, studies have begun to incorporate iNPH patients’ brain biopsies to study AD pathology-related mechanisms utilizing scRNA or snRNA sequencing [7, 43]. Indeed, early AD pathology in iNPH patients correlates with increased expression of microglia, astrocyte and neuronal genes [43]. The snRNA sequencing of iNPH brain biopsies has further revealed a transitional hyperactive state in upper cortical layer pyramidal neurons, preceding the loss of inhibitory and excitatory neurons [7]. Furthermore, a microglia-induced neuroinflammation is present throughout the AD continuum and in Parkinson’s disease [7]. Interestingly, Aβ production-associated genes were found to be upregulated in both oligodendrocytes and pyramidal neurons in brain biopsies presenting early AD pathology [7]. This phenomenon was further examined in an induced embryonic stem cell-derived model, which confirmed the ability of both cell types to produce Aβ. Regarding transcriptomics research, mouse models show partially different expression profiles in most disease-relevant microglia states of early AD, emphasizing the importance of studies based on human tissue [7].

#### Brain biopsy in the investigation of cell structure and function

A recent advancement in transcriptomics is the integration of snRNA sequencing data with cellular cytoarchitecture, known as spatial transcriptomics [11, 12]. This method is suitable with both fresh frozen and FFPE tissue samples [11]. Key advantages over sc- or snRNA sequencing alone include gaining additional cell location data in tissue and the ability to study cell–cell interactions and subcellular transcript changes [11]. This spatial information can be enhanced with additional genomic layers, such as epigenome, copy number variation, or expressed cell surface receptors, creating a multimodal spatial genomics approach [12]. The multi-omic approach can be further broadened by novel spatial proteomics that aim to identify selected protein abundances with spatial resolution [11]. The Slide-tags method in transcriptomics can achieve 10 µm spatial resolution in human tissue without interfering with snRNA sequencing, resulting in high-quality transcriptomic data suitable for identifying cell-type-specific, spatially varying genes in human brain tissue [12]. The significance of spatial transcriptomics is emphasized by findings that no notable functional differences were observed between postmortem tissue-derived microglia cultures from healthy individuals and those from AD, Lewy body disease, or frontotemporal dementia [92]. Hence, key microglial changes may only be detectable in proximity to brain pathology or affected neurons.

The obtained brain biopsy may be used for further investigation using techniques such as light and fluorescent microscopy, immunohistochemistry (IHC), densitometry, morphometry, and electron microscopy. IHC assessed with fluorescent and light microscopy reveals disease-related histological features and allows targeted immunoreactivity with selected antibodies. Densitometry and morphometry provide quantitative measurements of IHC staining reactivity or area, and can be enhanced using artificial intelligence (AI)-based analysis tools [93]. Electron microscopy further enables high-resolution imaging of cellular ultrastructure. In research involving living human brain biopsies obtained during neurosurgical procedures, these methods have been utilized to gain valuable insights into the glia-neuro-vascular interface [20, 94]. For example, iNPH patients have decreased expression of aquaporin 4 water transporters in their astrocytes based on IHC, densitometry, cytochemistry, and electron microscopy [20, 67, 94, 95]. Additional findings included increased cortical astrogliosis, fibrinogen extravasation, and decreased perivascular dystrophin (Dp71) expression in brain parenchyma [20, 67, 95]. Electron microscopy has also revealed a reduced number of normal mitochondria in astrocytic endfeet and a thinner capillary endothelial basement membrane [20]. These results suggest that blood–brain barrier dysfunction and the potential involvement of the glia-lymphatic system are involved in iNPH etiopathogenesis.

Human brain function arises from a network of diverse cells expressing complex interactions and adaptable connections. The CNS circuitry, structured by neurons and complemented by oligodendrocytes [3], is theorized to facilitate higher brain functions in humans [96, 97]. Several neurological and neurodegenerative diseases are often believed to involve disrupted or degenerating neurons as the underlying cause of their symptoms [98]. It is further acknowledged that rodent CNS neuronal circuits and dendrite excitability differ from those in humans [3]. Overall, most studies examining physiological or pathological neuronal functions are conducted in postmortem, cellular, and animal models that have significant limitations in translating knowledge to living humans [99]. However, researching such neuronal functions in humans in vivo, remains rare and is mainly limited to epilepsy patients undergoing multielectrode array (MEA) implantation during epilepsy-related surgeries [3], or to surrogate markers obtained via MRI [98, 100]. Ex vivo brain slice cultures provide an alternative platform for electrophysiological research, but limited availability of fresh samples and challenges in culturing have historically constrained their usability [3, 99]. Advances in culturing methods, however, have extended the viability of surgically obtained brain tissue from 12–72 h to several days or even weeks [3, 99]. These 3D cultures support electrophysiological recordings of neurons and can be genetically targeted through viral transduction, enabling research on disease-causing mutations and their effects on neuronal networks and cellular function [99]. Electrophysiological methods include whole-cell patch-clamp and MEA recordings. MEAs are advantageous due to their non-invasive nature, ability to detect extracellular potential changes and they require no manipulation of target cells with dyes [3]. In early AD, the upper layer excitatory neurons undergo a hyperactive state, as measured in fresh cortical tissue using 3D MEA [7]. Human induced pluripotent stem cell-derived genetic 2D and 3D neuronal models recorded with MEAs have further revealed that disease-associated *PSEN1* and *APPswe* alleles increase glutamatergic and GABAergic activity, cerebral mean firing rate, and synchronized bursts [3]. Moreover, the repeat expansion in the chromosome 9 open reading frame 72 (*C9ORF72*), a common cause for amyotrophic lateral sclerosis and frontotemporal dementia, affects motor neurons by increasing their firing rate in 2D models, but not in 3D organoids [3].

#### Challenges in postmortem, animal and cellular models

Research using postmortem or non-human brain tissue inevitably faces limitations. At the time of death, neurodegenerative disorders are often in advanced stages; furthermore, perimortem events can cause metabolic, infectious, and inflammatory changes in highly sensitive brain tissue that may mask disease-related differences. Meanwhile, animal models cannot fully replicate sporadic human neurodegenerative brain diseases due to genetic, epigenetic, physiological, and morphological variability [2, 7]. Autopsy-based cytological cultures, as disease models, can be further misleading or insufficient, as they fail to recreate the disease-like environment for cell populations and suffer from an inability to capture viable neurons for study [92, 101]. In neurodegenerative brain disorders, microglial immune responses are dependent on their surrounding environment [92]. The human pluripotent stem cell 2D cultures or organoids, on the other hand, mostly represent early developmental stages of the brain and lack complexity in cellular diversity and cytoarchitecture, which presents a challenge for lifetime generalizability [3]. Postmortem brain tissue experimental studies are often affected by the changes related to the agonal/hypoxia state at end of life, RNA and DNA degradation over time, and the period from death to neuropathological assessment, which may introduce confounding variability [7, 102, 103]. A recent study reported that the postmortem interval had no correlation to their gene expression analysis result involving neurons and astrocytes [102]. However, in another study, the postmortem interval degraded the RNA in inhibitory and excitatory neurons and increased artifact-related gene-expression in microglia [7]. A comparison between neocortical grey matter from epilepsy surgeries and autopsies [103] further found that the fidelity of transcriptional complexity is reduced in postmortem brain tissue. Moreover, a postmortem interval of a few hours led to a reduction in neuronal activity-dependent transcripts, while housekeeping genes were relatively well preserved [103]. In the first 24 h of the surgical sample resection, a counter-effect occurs, as astroglia- and microglia-related gene expression increases in the tissue [7, 103]. Therefore, high-quality living patient samples from target tissue, combined with timely and precise logistics until culture or sequencing, are likely essential for understanding the mechanisms of diseases at the cellular and tissue levels [2, 6].

Taken together, this literature indicates that brain biopsies from living patients can offer valuable insights into human neurophysiology and complex neurological diseases. Here, we outline a protocol to safely obtain and process a small brain biopsy suitable for diagnostic and research purposes.

### Protocol for an additional brain biopsy

#### Additional brain biopsy during surgical management of NPH

**Fig. 2:**
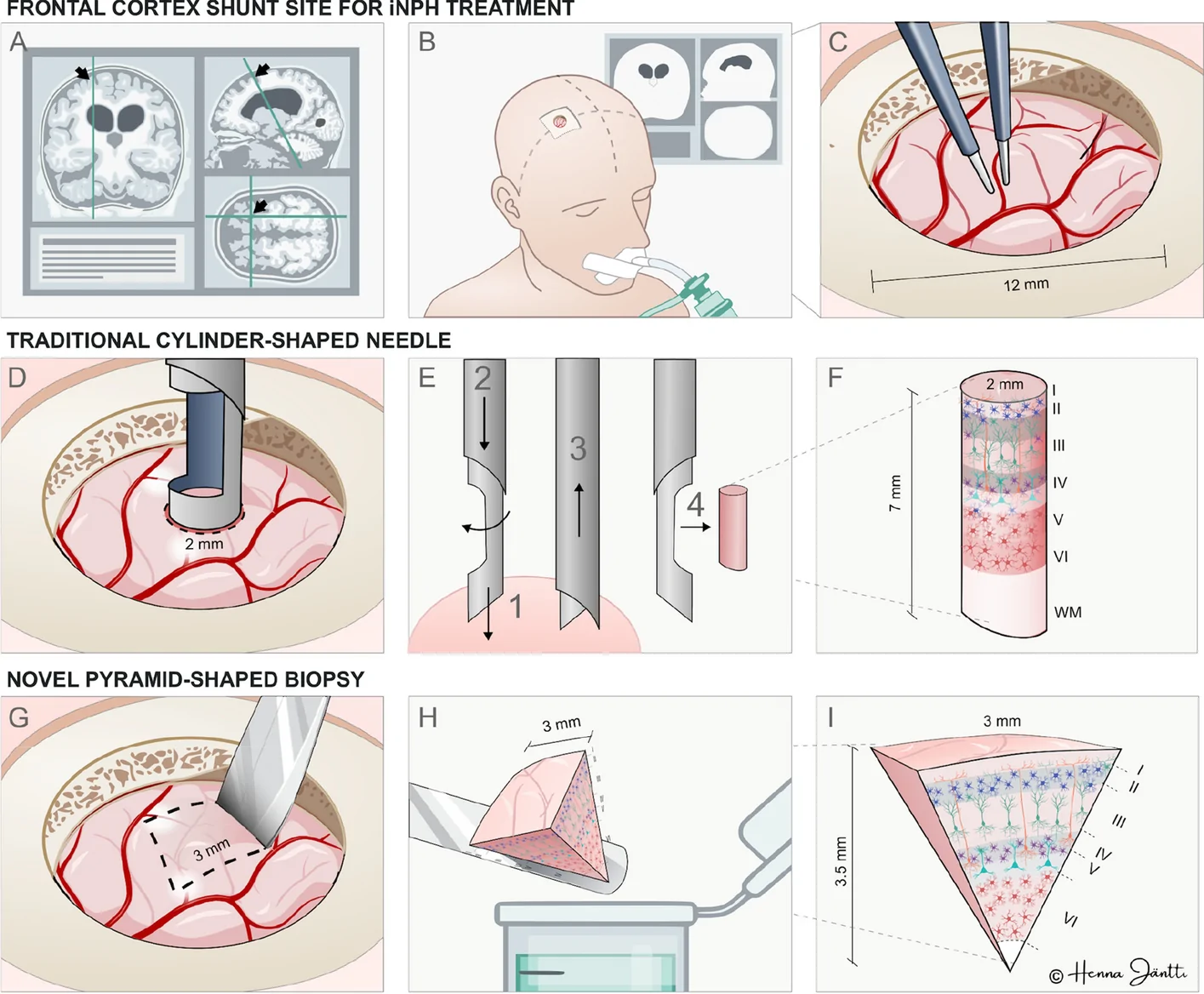
Kuopio brain biopsy acquisition protocols during iNPH shunt surgery. **A** Identify the biopsy site using CT or MR imaging. **B** Position the patient appropriately and locate the standard burr hole near the coronal suture along the mid-pupillary line for intraventricular catheter insertion. **C** Coagulate minor cortical vessels using bipolar forceps. Avoid sulci and note that bulky cortical veins should be preserved. If necessary, enlarge the burr hole to avoid damaging vessels. **D**, **E** & **F** Approach 1: Carefully applicate the biopsy tool on the surface of the targeted biopsy site. Obtain the sample using a stereotactic biopsy needle or a disposable biopsy tool. Rinse the intact biopsy into a cryotube. **G**, **H** & **I** Approach 2: Carefully excise an intact cortical brain biopsy with a sharp cut using a small surgical knife. Gently lift the pyramidal biopsy sample, approximately 9 mm^3^ in size, using a dissector and transfer it into ice-cold growth medium. Ensure that all cortical cell layers remain intact and viable in the sample

The NPH and Early AD – Research Group of the NeuroCenter of KUH and the Neurosurgery Department of University of Eastern Finland in Kuopio, Finland, has performed surgical brain biopsies for diagnostic and research purposes as part of standard clinical care for NPH since 1991 [7, 29, 32, 41, 45, 58, 60, 61, 64, 66, 73, 86, 87, 104]. In 2010, a similar approach for research brain sample collection was adopted by the Department of Neurosurgery at the University Hospital and the University of Oslo, Norway [20, 67]. Our research groups have developed two primary methods for obtaining brain biopsies. Method 1: Cylindrical brain biopsy using a biopsy tool. Method 2: Pyramidal brain biopsy via surgical excision. The cylindrical brain biopsy has been used clinically at the University Hospital of Oslo since 2018 and at KUH since 2010. In 2019, KUH’s iNPH protocol was revised to incorporate the current pyramidal cortical brain biopsy technique. In 2021, the surgical excision biopsy technique was also adapted in UCL Queen Square institute of neurology, London, UK. As a result, extensive experience with these methods has been accumulated. The characteristics of both approaches are summarized in Table 2, and the obtainment protocols are shown in Fig. 2. Before any CSF shunt insertion and cortical biopsy, eligible patients are evaluated by a multidisciplinary team consisting of a memory nurse, neuropsychologist, neurologist, neuroradiologist, and neurosurgeon following the previously described Kuopio iNPH protocol [105]. This protocol involves CT or MR imaging, routine blood samples, clinical assessment and lumbar tap test with CSF sampling. If a probable iNPH diagnosis is received and the patient is willing, the CSF shunt surgery is provided.

**Table 2:** Advantages and disadvantages of two surgical approaches to brain biopsy

Topic	Approach #1: Cylindrical	Approach #2: Pyramidal
Indication	+ Applicable for multiple neurosurgical procedures + Diagnostic and research use + Subcortical procedures	+ Applicable for multiple neurosurgical procedures + Diagnostics and research use - Only superficial procedures
Technique	+ Easy to learn + Quick + Stereotactic needle used - More difficult to control hemorrhage	+ Superficial approach + More controllable + No special equipment needed - Requires practice to obtain optimal samples - May be considered more invasive
Output	+ Neuropathological diagnosis + Morphological analysis + Genomics + Proteomics + Evaluation of deeper brain tissue ± Spatial analysis limited - Hard to obtain living cells	+ Same as cylindrical + Spatial analysis + Obtain living cells for electrophysiological recordings + Represents the cortex better
Risk aspects	+ Only minor additional risk for complications in shunt surgeries by literature - More difficult to avoid intracerebral minor blood vessels	+ More superficial and controllable technique may be less prone for complications + Better risk benefit ratio from more versatile use - Limited information for additional risk involved in procedure

legend: **(+)** Indicates the advantages of the method across different categories. **(-)** Indicates the disadvantage of the method across different categories

#### Indications for obtaining a brain biopsy

In general, each therapeutic intracranial neurosurgical procedure offers a potential opportunity for brain biopsies from non-eloquent cortical areas when examining neurodegenerative brain pathologies. Incorporating additional biopsy collection as part of standard neurosurgical treatment may be mutually beneficial by providing additional diagnostic value and enabling the acquisition of research samples. Currently, there are no standardized guidelines for obtaining brain biopsies specifically for neurodegenerative diagnostics or research, nor is this practice widely adopted in neurosurgical units worldwide.

Based on literature review and our clinical experience, frontal cortical brain biopsy during therapeutic intraventricular shunt catheter placement in iNPH carries minimal additional risk and has clinically meaningful implications for comorbidity diagnosis and prognosis. Therefore, we consider that an additional brain biopsy may have a clinical indication in selected cases, where a neuropathological diagnosis is expected to influence treatment and less invasive options are insufficient. In such cases, the decision to perform a biopsy should be based on thorough risk–benefit assessment and evaluated on a case-by-case basis. We recommend a multidisciplinary approach to risk–benefit assessment, the use of a standardized technique, and involvement of a neurosurgeon experienced with the procedure.

Furthermore, based on reasonable safety profile, the collection of an additional brain sample for research purpose may be considered in selected iNPH patients undergoing shunt surgery in experienced centers and under standardized protocols. Due to the novelty of this approach, prospective safety monitoring, regional approval by a regional ethics review board, and prior explicit informed consent are necessities. We also consider this recommendation to apply to neurosurgical procedures in which the anticipated additional risk of biopsy is comparable to that observed in iNPH shunt surgery. In other neurosurgical procedures beyond this definition – such as evacuation of intracerebral hemorrhage, tumor removal, or epilepsy surgery – the decision to perform an additional research brain biopsy should be evaluated only on a case-by-case basis, while adhering to the same ethical and safety monitoring principles.

#### Surgical techniques for a brain biopsy during iNPH shunt surgery

Patients are operated on under general anesthesia. The target site for the ventricular catheter is marked along the midpupillary line approximately 3 cm from the midline and anterior to the coronal suture, in the area of the non-dominant frontal lobe of the cerebral hemisphere (Fig. 2A & B) [105]. The aimed biopsy location therefore mainly represents Brodman areas 8 or 9 [7]. The skin is prepared and draped routinely. After revealing the superficial skull, a 12 mm burr hole is made using a trepan drill (Fig. 2C). The burr hole can be expanded using a bone punch for maximum exposure of the cortex. The dura mater is exposed and opened in a cross shape, gently lifted, and a dura sample for research purposes is obtained. The anatomical structures are identified. Biopsy is taken from the intended insertion site of the intraventricular catheter before insertion to prevent excessive impact on the brain tissue. If major venous structures cross the planned biopsy site, an alternative route is recommended. A larger burr hole and further dura exposure may be necessary. Careful planning with preoperative images, intraoperative skull landmarks, or neuronavigation helps to ensure precise location.

##### Approach #1: Cylindrical biopsy

Cylindrical brain biopsies can be obtained from the targeted ventricular catheter site if limited information is required from the sample (Table 2). A disposable biopsy tool (e.g., Temno Evolution TT146, Merit Medical Systems Inc, South Jordan, UT, USA) or a standard brain biopsy needle used in stereotactic procedures can be used to collect cylindrical biopsies measuring 1–2 mm in diameter and 2–8 mm in length (Fig. 2D-F) [105]. The needle of the stereotactic biopsy device should be stabilized with the device itself or by a neuronavigation arm. The needle has a blunt head and may be less traumatic, offering a gentler biopsy approach. Marked vessels should be avoided near the targeted biopsy site, and adequate hemostasis should be achieved both before and after using the biopsy tool.

##### Approach #2: Pyramidal biopsy

A pyramid-shaped cortical biopsy is obtained by making four precise cuts with a knife into the most superficial brain parenchyma. Each cut measures approximately 3.0 mm wide, 1.5 mm from the center of the targeted ventricular catheter insertion site, 3.0 mm deep, 3.4 mm long, and inclined at about 65° from the brain surface (Fig. 2G). Next, the biopsy is gently lifted using a dissector, resulting in an approximately 9 mm^3^ sample (Fig. 2H-I). To maintain cytological architecture, it is crucial to use a sharp blade and a gentle lift to minimize manipulation of the sample. Sharp dissection through the arachnoid can be enhanced with a diamond or needle knife if needed. Major vessels should be preserved, and minor vessels coagulated with bipolar forceps set to low power, as far from the biopsy site as possible (Fig. 2C). All bleeding, even minor, should be coagulated using bipolar forceps; rarely, hemostatic products are required. Blood contamination must be avoided at every step.

#### Surgical risks and quality of the biopsy

Obtaining additional brain tissue samples using sharp surgical techniques involves risks such as additional bleeding during the operation, postoperative intracerebral hemorrhage, that can lead to focal or generalized seizures, abscess or bacterial meningitis, subdural hematoma and hygroma, transient hemiparesis, ischemic stroke, and prolonged operation time [13, 14, 60]. Additional cylindrical cortical brain biopsies obtained during ICP recording in patients with suspected NPH at our center or collaborating institutions have resulted in biopsy-related serious adverse events in 6 (1.1%) cases of the total 551 cases, three (0.5%) of which ultimately led to death during a 12-month follow-up [20, 60, 67]. In our subsequent experience with a cohort of 1009 patients shunted for NPH, we obtained additional brain biopsies in 620 patients (Table 3). During the 12-month postoperative follow-up, we observed 3 and 0 shunt surgery-related serious adverse events leading to death in the additional biopsy and standard treatment groups, respectively. Two cases occurred acutely postoperatively and involved intracerebral hemorrhage; one of these cases also developed an infection. The third case experienced a prolonged epileptic seizure acutely and later developed a fatal shunt infection. All three cases were operated on using the cylindrical biopsy protocol. The resulting mortality rates were 0.48% (95% CI 0.01–1.40) for the biopsy group and 0% (95% CI 0–0.94) for the standard treatment group, and the difference between the rates was not significant (*p* = 0.29). All-cause mortality proportions were similar between groups (Table 3) (OR 1.04 [95% CI 0.6–2.0], *p* = 1). Group without brain biopsies were younger and had more females (Table 3). Based on these results, we consider additional brain biopsy to pose minimal additional risk to postoperative survival.

**Table 3:** Patient characteristics and postoperative mortality

		**With Brain biopsy**	**Without Brain biopsy**
		**(*n* = 620)**	**(*n* = 389)**
Age; mean (SD)		74.9 (6.7)	71.2 (8.3)^a^
Sex (M); n (%)		348 (56.1)	177 (45.5)^b^
Mortality, 12-months; n (%)			
	All-cause	30 (4.8)	18 (4.6)^c^
	Operation-related	3 (0.5)	0 (0)^d^

legend: Baseline characteristics and mortality proportions are presented and compared between the additional brain biopsy group and the standard shunt surgery group

^a^*p* < 0.001, t = −7.4

^b^*p* < 0.05

^c^*p* = 1

^d^*p* = 0.29

Moreover, the surgical approaches for both biopsy types are easy to learn and, with experience, require less than 5 min to complete. In our experience, however, obtaining cylindrical brain biopsies, compared to pyramidal ones, may more often result in minor bleeding during surgery. Therefore, pyramidal biopsies, being slightly more superficial, visible, and controllable, are potentially safer.

The overall quality relies on the systematic atraumatic obtainment and handling of the tissue. Additionally, preservation should be carried out immediately after obtainment [4]. For electrophysiological purposes, the most significant factors for the sample viability are the time interval from the operating room to the recording site and the prerecording preservation protocol [106]. For other purposes, the most important factors are rapid freezing, temperature-controlled storage at −70 to −80 °C, and choosing a biopsy site that includes a large amount of grey matter. To objectively evaluate the sample's suitability for electrophysiological research, we use Nissl staining for cryosections as described previously [7]. This allows the selection of samples with coherent grey matter from the pia to L5 cortical layer for further recordings.

#### The slicing of brain biopsy for multimodal studies

The preservation of the sample depends on the intended analyses. In our practice, cylindrical brain biopsies have been used in routine neuropathological analysis, research IHC, proteomics, and snRNA sequencing. The pyramidal brain biopsy, however, is more versatile (Table 2). We use it for electrophysiological examinations, histology, IHC, proteomics, and spatial transcriptomics. Here, we describe the preservation techniques required for each purpose of the brain sample.

After surgical retrieval, the pyramid biopsy is immediately placed into a sterile 50 mL polypropylene tube filled with ice-cold, oxygenated, and sterile-filtered artificial CSF [7, 106]. If endogenous fluorophores or photoluminescence will be investigated, the samples can be covered from light with folio or optically sealed casket to minimize photobleaching. The biopsy is then delivered to the neurophysiology laboratory within 15 min from excision and prepared for electrophysiological analysis following the protocol described by Gazestani et al*.* [7]. Any debris or blood clots on the cortex surface are gently removed by small brush. The biopsy is further photographed, embedded with agarose gel and sliced into 350 µm slices using a vibratome in chilled (2–4 °C), fully carbogenated aCSF [7].

The extremities of the sample are preserved for proteomics (first and last slices), and mid-sample mini slices (2 slices) are included for functional testing that requires additional recovery procedures. The other slices are used for snRNA sequencing (1 slice), IHC, and histology analysis for research purposes (1 slice, with subsequent cryosectioning to 20 µm slices, typically 12–15 good thick slices), as well as for diagnostics (1 slice). These slices are then fixed in 4% paraformaldehyde overnight, followed by fixation in 30% sucrose, and stored in a temperature-controlled freezer at −70 °C.

After cutting, the mid-sample slices designated for microelectrophysiology (MEA and patch clamp) require further preservation. We employ a two-step recovery protocol in recovery aCSF solution, as previously described [7, 104, 106], before use for electrophysiology experiments. For the electrophysiological analysis of living cells and network activity, the intact fresh slices after the section in ice-cold oxygenated aCSF should be immediately transferred into 37 °C aCSF for recovery and to ensure the viability of the functioning neurons [7, 104, 106]. The recordings should immediately follow the recovery protocol to ensure the highest sample quality. After recording neuronal and network activity, the slices are preserved for further analysis using IHC and histology to assess the condition of neurons and glial cells, as well as neuronal morphology [104].

When preserving cylindrical biopsies after surgical removal, they are first rinsed with physiological saline (0.9% NaCl solution) to remove them from the biopsy tool, then placed into 1.5 ml cryotubes. This minimizes biopsy handling and helps prevent blood contamination. The cryotubes are immediately transferred to a liquid nitrogen container, frozen, and stored at −80 °C. These frozen biopsies are suitable for later neuropathological analysis, snRNA sequencing, and proteomics. The fresh-frozen tissue is ideal for assessing the brain's transcriptomic profile. To maintain RNA integrity, the delay from extraction to freezing should be less than 15 min, ideally as short as possible. This can be reduced to under one minute by using a liquid nitrogen ewer in the operating room.

To further preserve a fresh frozen brain biopsy for snRNA sequencing, we use the following published protocol for cylindrical biopsies [7]. Briefly, fresh-frozen brain biopsies are cryosectioned at −15 to −20 °C into 60 µm sections and then placed on dry ice. Each sample is processed with Extraction Buffer, triturated, and eventually diluted with wash buffer before being split into four samples. The diluted samples are centrifuged, and the supernatant is carefully aspirated. The remaining nuclei pellets are then resuspended in wash buffer, triturated, and prepared for further snRNA sequencing.

Histological analysis and IHC for diagnostic and research purposes can be performed on both sample types. Before analysis, frozen brain samples are fixed in buffered formalin for 24 h. The samples are then embedded in paraffin and cut into 7 µm sections. The sections are deparaffinized, rehydrated and prepared for histological and immunohistochemical staining.

#### Applications in diagnostics and research

**Fig. 3:**
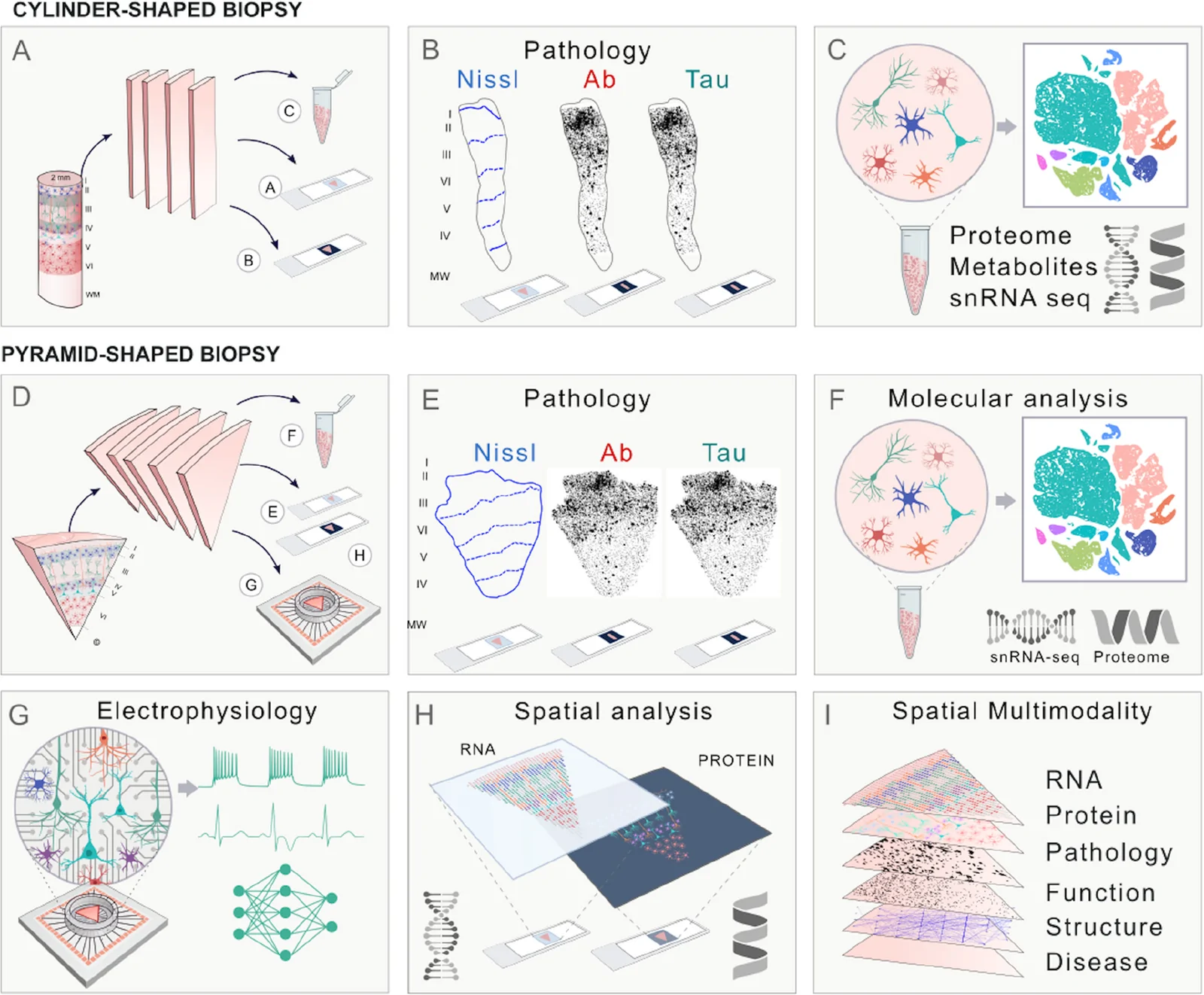
Applications of brain biopsy based on biopsy type. **A** Cut the 2 × 6–8 mm cylindrical biopsy for downstream applications of (**B**) diagnostic immunostaining, **C** proteomic and transcriptomic analyses. **D** Cut the pyramidal biopsy sample into 350 µm thick sections, suitable for (**E**) diagnostic and research immunostaining, **F** single cell/nuclei RNA (snRNA) sequencing and proteomics, **G** electrophysiological recordings and **H** spatial information integration. **I** The pyramidal brain biopsy offers a multimodal fresh tissue sample, enabling a wide range of research and diagnostic applications

Fresh brain samples are considered the most versatile for most research applications (Table 2). At KUH Neurosurgery, with our multidisciplinary and international research network, we have successfully used cylindrical biopsies for diagnostic neuropathology, and research purpose IHC, electron microscopy, proteomics, and snRNA sequencing (Fig. 3A-C). Pyramidal biopsies are also utilized for MEA and patch clamp recordings (Fig. 3D-H). The pyramidal biopsy includes tissue with overlaying leptomeninges, full-thickness cerebral cortex, and a small amount of subcortical white matter. The cylindrical biopsy contains pia and arachnoid mater and both grey matter of the cerebral cortex and subcortical white matter in varying amounts. For versatility, KUH Neurosurgery has increasingly transitioned to pyramidal-shaped brain biopsies, as they allow a wider range of research applications and are essential for adequate electrophysiological recordings. However, cylindrical biopsies remain a potential second option when living cells are not required.

The diagnostic neuropathological assessment is carried out by a neuropathologist (Fig. 3B & E). The preserved 7 µm sections are used, and hematoxylin–eosin stain, along with IHC with antibodies 6F/3D (M0872; Dako; dilution 1:100; pretreatment 80% formic acid 1 h) and AT8 (3Br-3; Innogenetics; dilution 1:30; pretreatment none) are applied to detect the presence of Aβ plaques and NFTs [60]. The readout is taken via light microscopy. The samples are also well-suited for applications involving AI-assisted analysis. Aβ plaque burden is semi-quantitatively recorded as mild, moderate or severe [28, 58]. Alternative options for assessing Aβ and NFT-pathologies include silver staining methods, such as Bielschowsky silver staining. We consider them to be valid alternatives; however, they are less sensitive to Aβ pathology than IHC [66]. The amount of transactive response DNA-binding protein-43 (TDP-43) is further recorded using IHC with monoclonal antibody 2E2-D3 (Abnova; dilution 1:1000; pretreatment autoclave in aqua, 120° and 80% formic acid 5 min). If needed, we complement the assessment with α-synuclein monoclonal antibody KM51 (Novocastra; dilution 1:1000; pretreatment autoclave in citrate buffer and 80% 120° formic acid 5 min) stain. The p62 (sequestosome 1) immunostain (clone 3; BD Biosciences Pharmingen; dilution 1:1000; pretreatment autoclave at 120° in 0.01 mol/l citrate buffer pH 6.0) is also added to samples to provide additional information on tau, α-synuclein, TDP-43 and polyglutamine protein deposits. These stains in relation to iNPH, provide a comprehensive screening of common primary and co-pathologies.

In research settings, standard histochemistry and immunostaining can be supplemented with other research and clinical IHC techniques if necessary. The assessment of immunocomplex levels using densitometry and morphometry may yield additional insights, as can AI-assisted detection and quantification of the pathologies [93]. Brain samples further hold the capability for cytochemistry stains and electron microscopy readings. These research methods provide tools for both basic research and the study of targeted disease mechanisms of clinical relevance. Furthermore, the surgical brain sample is usable for studying human electrophysiological CNS function (Fig. 3G). Our research teams have utilized the 3D MEA technique as described in Gazestani et al*.* [7] and patch clamp technique as described in Dougalis et al*.* [104]. However, fresh brain tissue samples are sensitive and require careful handling to preserve neuronal viability and the structure of the functioning neuronal network. In our experience, the pyramid-shaped brain biopsy, using the protocol presented here, is suitable for electrophysiology, whereas the cylindrical brain biopsy lacks cellular viability and neuronal network complexity. Transcriptomics by snRNA sequencing is also a powerful research method applicable to fresh human brain biopsies (Fig. 3C & F). In our experience, the samples preserved and analyzed using the protocol presented here and in Gazestani et al*.* [7] provide a comparable or higher quality transcriptome with marked granularity in cell identification and nuclei acquisition compared to studies using autopsy-based material. Transcriptomic analysis of brain biopsy samples can provide a direct readout of pathology-related cell state changes in the CNS and facilitate the direct correlation of CNS cell states with peripheral cell states. The transcriptome from brain biopsies can be further enhanced with spatial information about the sample, allowing researchers to investigate local transcriptome alterations. However, the cylindrical shape of brain biopsies may limit the spatial data obtainable due to their small diameter. The specific anatomical location of each biopsy can be determined retrospectively through postoperative CT or MRI scans. The location is typically identified from the ventricular catheter’s proximal entry point to the parenchyma, with the catheter’s midline offering the best estimate for accurate placement. The protocol for transverse, sagittal, and coronal measurements using multiplanar reconstruction with postoperative CT or MRI is detailed in the publication [7].

#### Clinical follow-up and additional samples for research

**Fig. 4:**
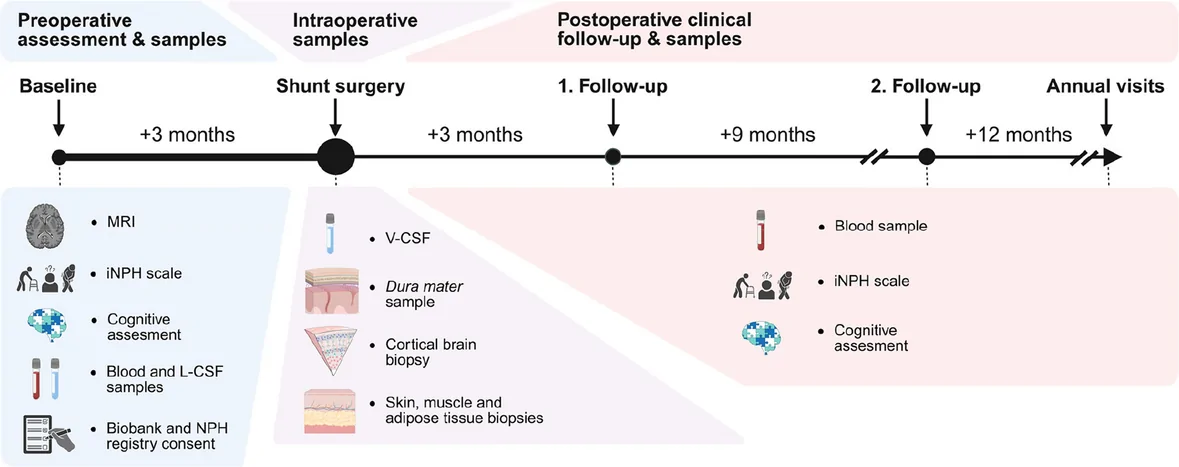
Kuopio University Hospital iNPH research sample protocol. The Neurosurgery of the NeuroCenter at Kuopio University Hospital has established a standardized research sample protocol for iNPH patients undergoing shunt treatment and participating in the iNPH Registry. Each eligible participant is managed according to the protocol outlined herein. iNPH, idiopathic normal pressure hydrocephalus; L-CSF, cerebrospinal fluid obtained by standard lumbar puncture; MRI, magnetic resonance imaging; V-CSF, cerebrospinal fluid obtained from ventricular catheter. Created with BioRender. https://BioRender.com/otzf7xr

At NeuroCenter of Kuopio University Hospital, we have implemented a research and sampling pipeline for patients with possible iNPH [107] to maximize the use of valuable living human samples. Each patient assessed for iNPH at the outpatient clinic is offered participation in the research protocol. Our tertiary medical center provides neurosurgical services to the entirety of Eastern Finland, with a catchment population of over 800 000 inhabitants. The research protocol has resulted in a regional cohort, which is maintained in the iNPH Registry and Biobank under our NPH and Early AD research group (www.uef.fi/nph). The recorded clinical parameters and types of samples obtained are shown in Fig. 4.

To create a patient-centered, multi-omic, and resourceful cohort for translational neuroscience, we have added several other parameters to our iNPH sample protocol. Core clinical symptoms and cognitive function are screened preoperatively and monitored annually after shunt surgery by CERAD neuropsychological battery and iNPH scale [108, 109]. MRI is performed during diagnostics, and blood samples for routine tests as well as biobanking are collected. A large volume of CSF (30–40 ml) is drained during the routine tap test assessment preoperatively [15]. Additionally, we collect small biopsy samples of dura, adipose tissue, muscle, and skin (for fibroblasts) during shunt surgery. These samples are preserved in the biobank and are easy to obtain during ventriculoperitoneal shunting, as all peripheral samples can be collected from the abdominal region when accessing the peritoneal cavity. Moreover, the dura sample is easily collected at the start of ventricular catheter insertion. Similarly, an additional CSF sample from the ventricles is obtained immediately after introducing the silicone catheter. At least 10 ml of CSF can be drained, allowing the surgeon to confirm the free flow of the intraventricular catheter. Overall, these multiple parallel samples provide a fundamentally precise cross-sectional view of iNPH and early AD. The clinical follow-up enables further comparison of individual longitudinal trajectories and outcomes in relation to baseline pathology. We present our current protocol to highlight the research opportunities created by neurosurgical treatment and to offer a feasible approach for high-quality human brain sample collection.

Similar but complementary research protocols have now also been established at other partner institutions such as the UCL Queen Square institute of neurology, where parietal biopsies are now routinely collected and pipelines that facilitate interrogation of protein structure and chemistry.

#### Ethical considerations and biobanking of research samples

All individuals suspected of having iNPH and being considered for therapeutic surgical treatment at KUH are potentially eligible study participants. Written informed consent is required prior to participation. If a patient has limited decision-making capacity, participation is discussed with the primary caregiver, and both the patient’s assent and proxy consent of the primary caregiver are required. Proxy consent must always align with the patient’s own will. Decision-making capability is evaluated on a case-to-case basis by neurosurgeon and is guided by CERAD-NB cognitive test results [108] and Clinical Dementia Rating score. Participation in the study, or a patient’s dissent for participation does not affect access to shunt surgery or any aspect of standard iNPH care. Participation is voluntary, and withdrawal of consent for research or Biobank participation is allowed at any stage of the study. Apart from the use of neuropathological diagnosis of brain biopsy, our research consent does not include returning incidental findings to patients. The research protocol has been approved by the regional ethics committee, the iNPH Registry operates under a data permit, and the organizational research permit is granted by the regional wellbeing county.

All the collected biological material not required for standard clinical care is stored in a biobank under Biobank consent. This consent enables broader research use of the samples and associated data and aligns with the objectives of the Finnish Biobank Act regarding future use [110]. Long-term storage and future use are managed by the Biobank organization. Access to pseudonymized data is also managed by the Biobank, and generally requires ethics review board approval, an explicit research plan, an independent biobank evaluation process, and a material transfer agreement before samples and data can be accessed.

## Discussion

Brain biopsy as a standalone diagnostic procedure in dementia with suspected or unknown etiology is uncommon and requires thorough non-invasive diagnostics and exclusion of other diagnoses before proceeding with it. However, in selected cases, a diagnostic brain biopsy can be valuable as it changes the management in about 30% of patients [48, 49]. Although, up to an 80% rate of changing the therapeutic orientation after brain biopsy has been reported in non-neoplastic retrospective cohorts [51, 52]. The clinical utility of living human brain biopsy is expected to grow when personalized disease-modifying therapies become available to neurological or neurodegenerative brain disorders [2, 6, 13, 60]. The thorough risk–benefit analysis should be conducted by a dedicated multidisciplinary team, with its neurosurgeons making the final decision to operate in mutual agreement with the patient and the caretakers.

An additional brain biopsy during iNPH or other neurosurgical procedures can be clinically beneficial for diagnostic purposes and may influence on future treatment plans. Standard neuropathological examination of the brain tissue often reveals whether a coexisting neurodegenerative disorder is present. Identifying such comorbidities guides dementia care, provides prognostic information, informs decisions about potential re-operations, and suggests options for medical treatment of disease or symptom progression [29, 61]. In iNPH patients, neuropathologically confirmed AD-related pathology provides clinically relevant insight into individual risk for future clinical AD. In addition, findings from the biopsy can help identify patients who could benefit from emerging neuroprotective therapies [28, 29, 36, 37, 39, 60]. Furthermore, alternative less invasive approaches, such as PET imaging and biomarkers, need further validation in longitudinal studies in iNPH to become as reliable predictors of comorbid AD pathology.

In human research, brain biopsies of adjacent white matter are uncommon but can be potentially obtained during elective epilepsy and brain tumor surgeries or acute evacuation of intracerebral hematomas, where surgical access to the target tissue passes through cortex and white matter [4, 5, 67]. However, neuroscience research still lacks more routine neurosurgical methods and ethical justification for obtaining living human brain samples [2]. Here we argue, based on current literature, our experience, and the presented protocol, that therapeutic surgery for NPH has the potential and feasibility to serve as a source of living human brain samples for research. The brain biopsy serves multiple purposes with proper acquisition and preservation, enabling a multi-omic novel research opportunity. Our clinical protocol enhances the translational potential of these samples, as repeated clinical assessments combined with additional tissue samples provide a broad patient-centered biobank for correlating individual clinical trajectories with pathological processes (Fig. 4). A similar sampling strategy is endorsed in glioma research by the international neuro-oncology consortium [4]. Moreover, neuropathological diagnosis is presumably advantageous to clinical diagnoses in neurodegenerative brain diseases and offers more detailed data for disease-specific comparisons [85]. The methodological protocol presented further aims to maximize the research benefits of these rare samples. We claim additional brain biopsies to be widely applicable for advanced research. A small cylindrical punch biopsy is suitable for snRNA sequencing, electron microscopy, and histochemistry. Moreover, analyses requiring intact cytoarchitecture and cellular viability will benefit from more refined techniques, such as our pyramidal approach, which includes a sharply excised 9mm^3^ pyramid-shaped biopsy. However, beyond the already reported RNA sequencing quality metrics and sample selection criteria, further studies are needed to systematically evaluate sample quality across acquisition steps and applications. Furthermore, any applications beyond standard neuropathological assessment should be noted as research benefits of biopsy and conducted only under an established research protocol, with ethical approval and explicit informed consent.

Importantly, performing an additional biopsy during therapeutic shunt surgery in iNPH patients can be considered substantially safe. Based on data from both ICP recordings and shunt surgeries, the short-term rate of clinically significant complications is below 1%, and the mortality rate is comparable to that expected in iNPH shunt surgery without an additional biopsy (Table 3) [34, 60, 64, 68]. However, there is still a need for large-scale observational studies comparing all adverse events associated with additional small cortical brain biopsy during shunt surgery with those of standard therapeutic care for NPH. This limitation should be acknowledged when considering the recommended approaches. Lesions observed on MRI with precise location are beyond the scope of this paper. In such cases, the use of neuronavigation or stereotactic guidance is essential for accurate sampling.

A notable limitation is that the frontal cortical sample described here represents only a small, localized brain region, which should be weighted for the expected location of the pathological changes found in postmortem evaluation or molecular imaging. This dependency is well illustrated in simulated brain biopsies by Venneti et al*.*, where sampling from four regions improved both sensitivity for CBD, FTLD-TDP, LBD, MSA, PSP and PiD, as well as specificity for controls [54]. A similar limitation may be expected for biopsy in atypical AD cases [78]. Although presumed disease prevalence heavily affects post-test probability, lower sensitivity reduces the negative predictive value and therefore limits the ability to exclude AD, LBD, LATE, CBD, FTLD-TDP, vascular pathology or other neurodegenerative disorders based solely on biopsy finding. Moreover, the positive predictive value of biopsy, remains suggestive for neurodegenerative disorders other than AD. This is mainly driven by low expected prevalence, despite relatively high specificity. In the typical AD pathological cascade, even a small frontal (or parietal) cortical sample is representative of Aβ pathology, however, tau pathology appears in these regions only at advanced Braak stages [55, 56, 73]. Considering this, an A + T- biopsy alone has limited positive predictive value for clinical AD but is specific to at least early-stage AD-related pathological change. An A + T + biopsy, on the other hand, is expected to have substantially higher PPV for clinical AD due to its high specificity and the expected notable prevalence. Nonetheless, evidence from neuro-oncology shows that even a 1mm^3^ sample of viable tissue can be sufficient for advanced molecular pathology diagnostics [4], supporting the broader applicability of small brain biopsies in other clinical and research contexts.

A subset of iNPH patients carry AD-related pathology without clinical AD, which allows investigating the initiating disease mechanisms and targeted early disease drug treatments [39, 43]. Patients with iNPH also rarely present with amyotrophic lateral sclerosis and frontotemporal dementia [111]. If brain biopsies are obtained during shunt procedures, these samples could serve as valuable groups for studying these conditions as well. The advantage of studying the AD-related disease mechanisms in living iNPH patients is not only the ability to observe various stages of AD pathology (Fig. 1), but also the chance for long-term or lifelong clinical monitoring and potential postmortem confirmation to link with earlier findings [28, 36, 39, 43]. Unlike postmortem studies, all clinical parameters and parallel samples can be collected during the same visit, removing time gaps between variables. Therefore, iNPH patients offer unique access to living human brains with and without AD-related pathologies to support early AD research. Additionally, iNPH brain biopsies obtained with the described techniques are of high-quality for transcriptomics, offering a high number of cells sampled per cell type and minimal artifact-related gene expression compared to postmortem human and mouse samples [7]. Recent advances in culturing the brain biopsy slices in vitro further allow examination of neuronal function, morphology and neuronal networks [99]. These tissues can also be genetically modified via vector transduction, creating a viable model for investigating disease-specific genes [99]. This provides precise, human-based insights into disease mechanisms and allows exploration of how genetic background influences neuronal function.

The 2D or 3D organoid cell cultures from human induced pluripotent stem cells can serve as simple physiological models or drug testing platforms and they are capable of expressing the desired genome [3]. This especially allows for studying the physiological impact of significant disease-associated genes [3]. Both platforms, compared to in vivo MEA recordings, act as a continuum by measuring similar properties with increasing complexity. This allows downstream research to explore disease-associated pathophysiology in rare in vivo models and to structure suitable organoids for further targeting of novel drug and genetic therapies.

An ongoing question in iNPH and its comorbidity research is the generalizability of findings to non-iNPH-diagnosed disease populations. Recent literature has shown that isolated neurodegenerative pathologies are relatively uncommon in clinically diagnosed neurocognitive diseases. The same applies to iNPH as well, as most patients exhibit a second brain pathology, most often AD or vascular brain pathology [27, 28]. Gazestani et al*.* [7] compared gene expression patterns of different CNS cell types between iNPH patients with AD and postmortem non-iNPH AD populations, finding highly concordant changes between these populations. This strongly supports a similar ongoing disease process in both populations. Moreover, iNPH patients with IHC-confirmed AD pathology often develop clinical AD in line with the AD continuum hypothesis. However, it has long been debated whether these diseases share a common pathogenic pathway [112–114]. The literature is somewhat unclear from this perspective, and no causal link confirms iNPH as a subtype of other neurodegenerative disorders. At most, iNPH may serve as a risk factor for AD, and comorbid AD may potentially impair long-term cognitive outcomes [36, 37, 62–64]. Notably, short-term outcomes in iNPH appear unaffected by biopsy findings [33, 63] supporting this notion. In fact, there is growing evidence from genetic [23, 44, 45, 115–119], neuropsychological [120] and neuropathological studies [60, 61] as well as from a small randomized controlled trial with shunting [114] that highly corroborates the existence of iNPH as independent disease apart from AD or vascular cognitive impairment. Familial clustering of iNPH and associated genetic variants further reinforce this [115]. Furthermore, the iNPH GWAS identified four iNPH-specific risk loci [23] that, apart from those near *C16ORF95*, have not been associated with AD or AD-related dementias in previous GWASs [121–123]. Interestingly, three of these loci may nonetheless influence AD-related CSF biomarker levels [124]. From a neuropathological perspective, there are rare cases of patients who develop iNPH-related dementia with no known explanatory brain pathology observed in brain tissue [27]. These individuals are probably among the rare “pure” iNPH patients, corroborating the individual etiology. Neuropsychologically, iNPH patients show an individual cognitive decline profile on the CERAD-NB cognition test battery, differing from AD [120]. In addition, CSF shunting of AD patients does not improve their symptoms [125]. Furthermore, the discovery that extensive AD-related brain pathology in iNPH patients may hinder surgical outcomes supports the idea of AD as a true comorbid disease. Since advanced AD affects gait, continence, and cognitive functions, it is reasonable to expect that improvements in these symptoms are limited in such patients. Collectively, iNPH patients with AD represent an important study population for AD-related disease mechanisms. The relevance of these findings is further strengthened by longitudinal follow-up, which allows for the interpretation of outcomes in relation to shunt function or other iNPH-related events. However, the generalizability of these results to non-iNPH AD populations requires validation in independent cohorts. Due to ethical concerns, biopsy-derived findings will likely need validation through autopsy studies or biomarker-based surrogate measures. Nonetheless, the approach outlined here provides a more practical pathway for capturing early-stage pathological mechanisms of neurodegenerative brain diseases. After identifying potentially key mechanisms, further replication and validation can be conducted in smaller, targeted AD populations.

In the future, obtaining additional brain biopsies could be recommended in neurosurgical procedures where the presumed increased risk from the biopsy is considered low, and the diagnostic, prognostic, or research benefits justify their ethical acquisition. Having a research protocol that aligns both clinical and research objectives can help meet ethical standards. This approach not only allows the collection of valuable research disease-related samples but could also provide prognostic information to improve patient care or lead to modifications in the treatment plan. The importance of high-quality in vivo brain biopsies is emphasized for future multi-omic studies that have the potential to transform the treatment of various neurological and neurodegenerative brain diseases. Moving forward, we should look far into the future to anticipate the expectations that must be met by optimizing the biobanking of appropriate samples. We also advocate for cross center international collaboration to maximize research opportunities and to ensure best and safest clinical research practice.

## Conclusions

Thriving personalized patient care in neurodegenerative and neurological brain diseases requires significant translational research efforts and the development of high-quality databases. Parallel human tissue-based samples with longitudinal follow-up of patients are essential for achieving this. Patients with iNPH and additional research brain biopsies obtained during standard surgical management provide a unique, low-risk, and ethically acceptable approach to studying AD pathology across its entire spectrum and disease course. A surgically feasible technique involves atraumatic separation and handling of the brain samples, and preservation requires a precise protocol to maintain tissue quality.

## Data Availability

All data supporting the findings within this article will be shared anonymized by request of any qualified investigator.

## Abbreviations

A+: Amyloid-β pathology positive
Aβ: Amyloid-β
Aβ42: Amyloid-β 42
aCSF: Artificial cerebrospinal fluid
AD: Alzheimer’s Disease
AI: Artificial intelligence
ALS: Amyotrophic lateral sclerosis
CBD/MAPT: Corticobasal degeneration/microtubule-associated protein tau gene
CERAD: Consortium to Establish Registry for Alzheimer’s Disease
CNS: Central nervous system
CSF: Cerebrospinal fluid
CT: Computed tomography
CVD: Cerebrovascular disease
EEG: Electroencephalogram
FFPE: Formalin-fixed paraffin-embedded
FTLD-TDP/MND: Frontotemporal lobar degeneration with TDP-43 pathology/with motor neuron disease
GWAS: Genome-wide association study
HD: Huntington’s disease
ICD-10: International Classification of Diseases, 10th revision
IHC: Immunohistochemistry
INPH: Idiopathic normal pressure hydrocephalus
LATE: Limbic-predominant age-related TDP-43 encephalopathy
LBD: Lewy-body disease
MCI: Mild cognitive impairment
MCP1: Monocyte chemoattractant protein 1
MEA: Multielectrode array
MRI: Magnetic resonance imaging
MSA: Multiple system atrophy
NFT: Neurofibrillary tau tangles
NPV: Negative predictive value
P-tau: Phosphorylated tau
PIB-PET: Pittsburgh compound B positron emission tomography
PiD: Pick’s disease/behavioral variant of Frontotemporal dementia
PPV: Positive predictive value
PSP: Progressive supranuclear palsy
scRNA-seq: Single cell RNA sequencing
snRNA-seq: Single nucleus RNA sequencing
SUVR: Standardized uptake value ratio
TDP-43: TAR DNA-binding protein 43
T+: Tau pathology positive
T-tau: Total tau
